# Beta 2 Adrenergic Receptor Selective Antagonist Enhances Mechanically Stimulated Bone Anabolism in Aged Mice

**DOI:** 10.1002/jbm4.10712

**Published:** 2022-12-27

**Authors:** Leah E. Worton, Sundar Srinivasan, DeWayne Threet, Brandon J. Ausk, Phillipe Huber, Ronald Y. Kwon, Steven D. Bain, Ted S. Gross, Edith M. Gardiner

**Affiliations:** ^1^ Department of Orthopaedics & Sports Medicine University of Washington Seattle WA USA

**Keywords:** BONE‐BRAIN‐NERVOUS SYSTEM INTERACTIONS, EXERCISE, OSTEOBLASTS, PRECLINICAL STUDIES, TRANSCRIPTION FACTORS

## Abstract

The anabolic response of aged bone to skeletal loading is typically poor. Efforts to improve mechanotransduction in aged bone have met with limited success. This study investigated whether the bone response to direct skeletal loading is improved by reducing sympathetic suppression of osteoblastic bone formation via β2AR. To test this possibility, we treated aged wild‐type C57BL/6 mice with a selective β2AR antagonist, butaxamine (Butax), before each of nine bouts of cantilever bending of the right tibia. Midshaft periosteal bone formation was assessed by dynamic histomorphometry of loaded and contralateral tibias. Butax treatment did not alter osteoblast activity of contralateral tibias. Loading alone induced a modest but significant osteogenic response. However, when loading was combined with Butax pretreatment, the anabolic response was significantly elevated compared with loading preceded by saline injection. Subsequent studies in osteoblastic cultures revealed complex negative interactions between adrenergic and mechanically induced intracellular signaling. Activation of β2AR by treatment with the β1, β2‐agonist isoproterenol (ISO) before fluid flow exposure diminished mechanically stimulated ERK1/2 phosphorylation in primary bone cell outgrowth cultures and AKT phosphorylation in MC3T3‐E1 pre‐osteoblast cultures. Expression of mechanosensitive *Fos* and *Ptgs2* genes was enhanced with ISO treatment and reduced with flow in both MC3T3‐E1 and primary cultures. Finally, co‐treatment of MC3T3‐E1 cells with Butax reversed these ISO effects, confirming a critical role for β2AR in these responses. In combination, these results demonstrate that selective inhibition of β2AR is sufficient to enhance the anabolic response of the aged skeleton to loading, potentially via direct effects upon osteoblasts. © 2022 The Authors. *JBMR Plus* published by Wiley Periodicals LLC on behalf of American Society for Bone and Mineral Research.

## Graphical abstract

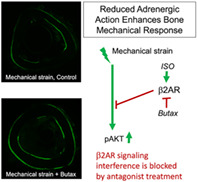

## Introduction

The anabolic response of bone to skeletal loading declines with age.^(^ ^1^ ^)^ Numerous age‐related bone cell deficits have been identified, including diminished osteoblast function^(^ ^2^, ^3^ ^)^ increased osteocyte apoptosis,^(^ ^3^ ^)^ a reduced osteoprogenitor pool,^(^ ^4^ ^)^ fewer periosteal lining cells,^(^ ^5^ ^)^ and a decreased calcium response to fluid flow.^(^ ^6^ ^)^ Additionally, systemic inhibitors are also likely to contribute to age‐related mechanotransduction deficits (eg, elevated serum sclerostin^(^ ^7^ ^)^). Despite these insights, the barriers preventing a robust osteogenic response from modest exercise in the elderly have not been overcome.

Anatomical studies reveal abundant sympathetic innervation in the periosteum of rodent long bones,^(^ ^8^ ^)^ with tyrosine hydroxylase (TH) immunoreactive fibers associated with blood vessels^(^ ^9^ ^)^ in the outer portion of the cambial layer.^(^ ^10^ ^)^ TH‐positive sympathetic nerve fibers have also been detected within diaphyseal cortical bone using fluorescent reporter mice.^(^ ^11^ ^)^ Aging does not alter the general morphology and organization of TH‐positive sympathetic innervation in mouse femora, but periosteal thinning in aged bone leads to increased sympathetic density despite a decrease in TH‐positive fiber numbers.^(^ ^12^ ^)^ Numerous other aspects of sympathetic regulation are also altered by age, including elevated plasma catecholamine,^(^ ^13^, ^14^, ^15^, ^16^, ^17^, ^18^ ^)^ sympathoadrenal changes,^(^ ^19^, ^20^, ^21^ ^)^ increased central sympathetic drive,^(^ ^16^, ^17^, ^18^, ^22^, ^23^, ^24^, ^25^, ^26^, ^27^, ^28^, ^29^, ^30^ ^)^ impaired peripheral clock entrainment due to sympathetic dysfunction,^(^ ^31^ ^)^ and increased norepinephrine content in cortical bone.^(^ ^11^ ^)^ In this context, and given our interest in cross‐talk between bone, muscle, and nerve,^(^ ^32^ ^)^ we posited elevated sympathetic tone in the aged as a potential underexplored contributor to the diminished osteoblastic response to skeletal loading in the aged.

We therefore sought to directly investigate the role of the β2AR in the in vivo osteoblastic response to skeletal loading and the in vitro response to fluid flow by transient pharmacological blockade using the β2AR‐selective antagonist Butax. This drug was chosen for the present study based on its augmentation of osteoblast activity in hypertensive rats^(^ ^33^ ^)^ and its low penetrance at the blood‐brain barrier.^(^ ^34^ ^)^ Specifically, we evaluated the osteoblastic response of aged wild‐type C57BL/6 mice to tibia cantilever bending treatment. In addition, we characterized signaling responses to fluid flow stimulation of mouse primary osteoblast outgrowth and pre‐osteoblast MC3T3‐E1 cell line cultures with and without β‐adrenergic receptor agonist treatment.

## Materials and Methods

### Animals

All experiments and protocols were approved by the University of Washington Institutional Animal Care and Use Committee (IACUC protocol no. 3306‐02) and were performed in accordance with the National Institutes of Health (NIH) “Principles of Laboratory Animal Care” guidelines (NIH publication No. 96‐23, 1996). Aged female C57BL/6 N mice (21 months) were obtained from the NIH/National Institute of Aging aged rodent colony (Charles River Laboratories, Wilmington, MA, USA). All animals were group‐housed in a SPF vivarium and acclimatized to local conditions (22°C room temperature, 14‐hour light /10‐hour dark cycle) for at least 1 month before experimentation and permitted standard commercial mouse chow and water *ad libitum* throughout.

### Chemicals and drugs

Isoproterenol (cat. no. I5627), butaxamine HCl (cat. no. B1385) and calcein (cat. no. C0875) were purchased from Sigma‐Aldrich (St. Louis, MO, USA). ISO stock was prepared fresh on each treatment day. Butax master stock (12.5 mg/mL in sterile physiologic saline) was stored in aliquots at −20°C and freshly diluted in sterile saline as needed. Tissue culture medium and supplements were purchased from Life Technologies (Thermo Fisher Scientific, Waltham, MA, USA). Media was supplemented with heat‐inactivated FBS (HyClone; Thermo Fisher Scientific).

### Imaging

Before experimentation, high‐resolution μCT images of the right tibia were obtained for all mice (Scanco vivaCT40; Scanco Medical, Bruttisellen, Switzerland; 10.5 μm voxel size, 55 kVp, 145 μA), and animal‐specific longitudinal normal strains induced by the loading protocol were determined via beam theory using individualized midshaft morphology.^(^ ^35^ ^)^

### In vivo loading protocol and tissue collection

Mice were randomly assigned to groups (*n* = 8/group) for treatment with saline or Butax (1.0, 3.0, or 10.0 mg/kg, i.p.). Experiments were conducted between 12 p.m. and 6 p.m. on Mondays, Wednesdays, and Fridays for 3 weeks (9 bouts total). Animals were weighed on each loading day, then acclimated quietly for at least 1 hour in covered cages to reduce stress. Each mouse received Butax or saline treatment, and 30 minutes later was anesthetized (0.2% isoflurane) and the right tibia loaded in cantilever bending using a noninvasive murine tibia‐loading device.^(^ ^36^ ^)^ Each loading intervention consisted of 50 cycles (1 Hz) calibrated to induce peak longitudinal normal strain of 1700 με at the tibia midshaft. All mice received calcein (10 mg/kg, i.p.) on days 10 and 19, then were euthanized on day 22. Two mice (one each from saline and 10 mg/kg groups) were euthanized and excluded from analyses when excessive weight loss was detected, per IACUC protocol. Loaded (right) and contralateral (left) tibias were dissected free of soft tissue and 500‐μm‐thick sections were obtained at the location where strain measurements were calculated. Sections were then hand‐ground to 125 μm, cover‐slipped, and imaged via epifluorescence microscopy (Zeiss Axio Imager.M2 epifluorescent microscope, Zeiss, White Plains, NY, USA). As previously described,^(^ ^37^ ^)^ composite images were assembled, anatomically oriented, and blinded for subsequent analysis. Custom NIH ImageJ based software was used to quantify mineral apposition rate (MAR, μm/d), mineralizing surface (MS/BS%), and bone formation rate (BFR, μm^3^/μm^2^/d) at the periosteal surface.

### RNA preparation and quantitative RT‐PCR

Total RNA was extracted from cultured cells using TRIzol (Life Technologies, Carlsbad, CA, USA). cDNA was synthesized using Superscript III reverse transcriptase (Life Technologies), and quantitative RT‐PCR was performed using SYBR green and the Applied Biosystems (Carlsbad, CA, USA) ViiA7 sequence detection system. Primer sequences are shown in Supplemental Table S1. Relative gene expression levels were quantified using the 2^(−ΔΔCT)^ method using β‐actin as housekeeping gene.

### Cell culture and fluid flow

Primary outgrowth bone cultures were prepared from marrow‐depleted tibial diaphyses of aged (22 months) female C57BL/6 mice. Bones from groups of 5 animals were pooled in two independent experiments. Cells that grew out of bone fragments in osteogenic culture medium (α‐MEM with 10% FBS, 2 mM L‐glutamine, 10 mM β‐glycerophosphate, 50 mg/mL ascorbic acid, and 100 U/mL penicillin/streptomycin) were expanded and then seeded at passage 2 for experimentation. MC3T3‐E1 cells (subclone 14; ATCC [Manassas, VA, USA] cat. no. CRL‐2594) were cultured in α‐MEM with 10% FBS, 2 mM L‐glutamine, and 100 mM sodium pyruvate. For cell mechanical stimulation, we used a previously described in vitro fluid flow model of mechanotransduction.^(^ ^38^ ^)^ Seventy‐two hours before experimentation, cells were seeded at 2.5 × 10^4^ cells/well into 6‐well plates in 2 mL of growth media. Eighteen hours before ISO treatment, cells were changed into growth media containing 0.5% FBS. On the day of flow exposure, cells were treated with ISO or Butax and transferred to an orbital shaker (VWR [Radnor, PA, USA], DS‐500) placed in an incubator at 37°C and 5% CO_2_, 30 minutes before exposure to fluid flow. Cells were subjected to orbital shaking (2.2 Hz, 0.1–0.5 Pa) and harvested for RNA after 1 hour of fluid flow or for protein analysis at 5 or 10 minutes after the start of flow.

### Antibodies and immunoblotting

Total protein was isolated from cells in RIPA lysis buffer (50 mM Tris–HCl pH 8, 150 mM NaCl, 1% NP‐40, 0.5% sodium deoxycholate, 0.1% SDS) and quantitated using BCA protein assay reagents (Thermo Fisher Scientific), then separated on 4–12% NuPAGE Bis‐Tris gels (Life Technologies) and transferred to Immobilon‐FL membrane (MilliporeSigma, Burlington, MA, USA). Blots were stained using REVERT Total Protein Stain solution (LI‐COR, Lincoln, NE, USA) and probed with primary antibodies at 4°C overnight. Antibodies are detailed in Supplemental Table S2. Bound primary antibodies were detected using DyLight 680 and 800 labeled secondary antibodies (Thermo Fisher Scientific, 1:15,000) and scanned and quantified using the Odyssey (LI‐COR). Where possible, phospho‐ and total protein antibodies from different host species were used at the same time to image dual fluorescent signals. When not possible, or to examine other signaling proteins, blots were stripped with Restore Fluorescent Western Blot Stripping Buffer (Thermo Fisher Scientific) and then reprobed. Quantified values for each signaling molecule on individual membranes were normalized to the associated no‐flow, no‐ISO value at the earliest time point on that membrane.

### Statistical analysis

Sample size for in vivo studies was determined by power analysis for a 20–40% difference,^(^ ^39^ ^)^ assuming α = 0.05 (based on results from our previous studies^(^ ^40^ ^)^ and the literature^(^ ^41^ ^)^). All data analyses were performed in R (http://www.R-project.org/). Mouse group weights and loading‐induced peak strains were analyzed via ANOVA. Body weight changes over the experiment were evaluated per group by repeated measures ANOVA. Non‐parametric statistics were used for histomorphometric assessment of bone response to mechanical loading. Kruskal–Wallis tests with Mann–Whitney follow‐ups (where appropriate) were used to determine if outcome measures differed across groups, and Wilcoxon's rank‐sum tests to contrast outcomes in loaded versus contralateral bones. Quantified Western blot and RT‐PCR data from in vitro experiments were analyzed via two‐ or three‐way ANOVA as indicated, with Bonferroni post hoc tests implemented as appropriate. Statistical comparisons are indicated in the figure legends. For all tests, *p* ≤ 0.05 was considered to be statistically significant.

## Results

### Butax pretreatment of aged mice enhanced the osteoblastic response to skeletal loading in aged mice

**Fig. 1:**
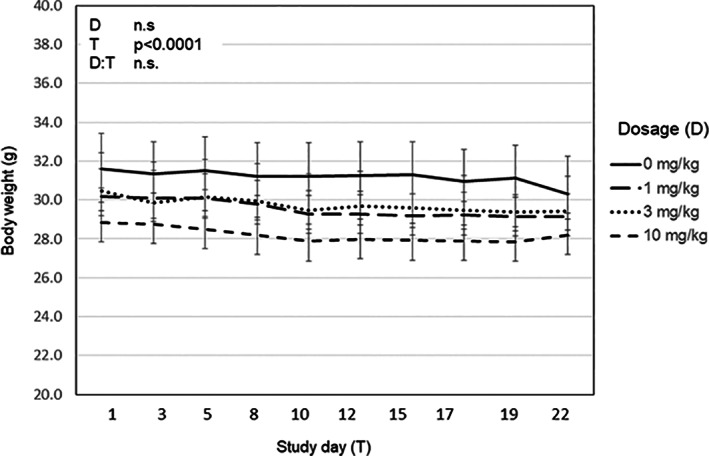
Butax treatment did not alter the decline in body mass in senescent mice. Mice exhibited a decline in body weight over the course of the study (*p* < 0.0001), but dosage had no effect (*p* = 0.78) and the interaction between study time course and dosage was not significant (*p* = 0.51).

**Fig. 2:**
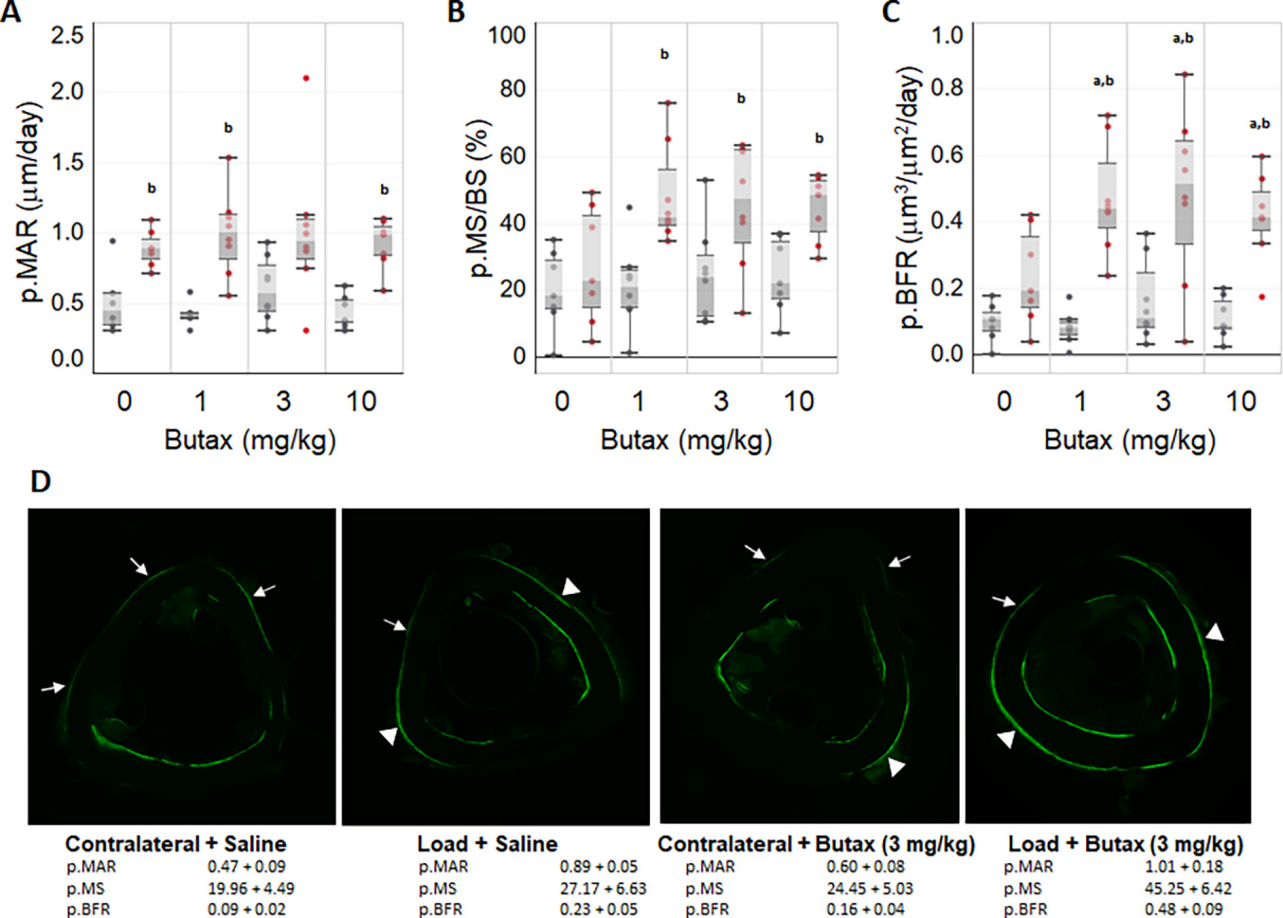
Butax treatment enhanced mechanically induced bone formation across a range of dosages. Periosteal mineral apposition rate (MAR) (*A*), mineralizing surface (MS) (*B*), and bone formation rate (BFR) (*C*) are plotted for loaded (red dots) and contralateral bones (black dots). Representative images (*D*) of contralateral and loaded tibias from saline and Butax‐treated mice (3 mg/kg dosage), each approximating the mean p.BFR of the specified group. Corresponding group mean values are listed below images. Single‐ and double‐labeled periosteal surfaces are indicated by arrows and arrowheads, respectively. Graphed data are from *n* = 7–8 mice in the 0, 1, 3, and 10 mg/kg (low, medium, and high) dose groups, respectively. Kruskal–Wallis test comparison across dosages for each limb identified significant dosage effect on p.BFR of loaded limb (*p* < 0.03). Mann–Whitney test compared each Butax dosage versus saline for this parameter (*p* < 0.05; ^a^Butax versus saline, same limb). Wilcoxon's rank‐sum test contrasted outcomes in loaded versus contralateral bones (*p* < 0.05; ^b^loaded versus contralateral limb).

We used in vivo skeletal loading and dynamic histomorphometry to examine the influence of Butax treatment on the anabolic response to mechanical loading. Baseline body mass and peak normal strain induced in the loaded (right) tibias did not differ across experimental groups (Table 1). Body weight significantly declined 3.3 ± 0.7% over the course of the study (*p* < 0.0001), but Butax dosage had no effect (*p* = 0.78) and interaction between study time course and dosage was not significant (*p* = 0.5; Fig. 1). No significant differences in contralateral tibias p.MAR, p.MS., or p.BFR were observed across experimental groups. When contrasted with contralateral tibias, p.MAR was significantly elevated by skeletal loading in the saline (0 mg/kg, *p* = 0.02), low‐dose (1 mg/kg, *p* = 0.01), and high‐dose (10 mg/kg, *p* = 0.02) Butax groups (Fig. 2). In contrast, p.MS was significantly elevated versus contralateral tibias only in Butax‐treated groups (low dose, *p* = 0.02; medium dose [3 mg/kg], *p* = 0.01; high dose, *p* = 0.03; saline, *p* = 0.47). Finally, p.BFR was significantly increased in the loaded tibias of each Butax‐treated group versus contralateral tibias (low dose, *p* = 0.01; medium and high doses, each *p* = 0.02), but not in the saline group (*p* = 0.22). As a result, p.BFR was significantly increased in the loaded tibias of the low‐ (*p* = 0.01), medium‐ (*p* = 0.04), and high‐dose (*p* = 0.04) Butax‐treated groups versus the loaded tibias of saline‐treated mice, but no differences were observed across the Butax dose range.

**Table 1:** Baseline Weights and Strain Values Did Not Differ Among Dosage Groups

Butax dose (mg/kg)	0	1	3	10
Body mass (g)	31.6 ± 1.8	30.2 ± 1.4	30.5 ± 2.0	28.8 ± 1.1
Peak normal strain (με)	1886.4 ± 73.6	1842.8 ± 84.5	1876.5 ± 101.3	1883.3 ± 72.8
Sample no.	7	8	8	7

### β2AR activation alters early mechanotransduction signaling in primary outgrowth bone cells

**Fig. 3:**
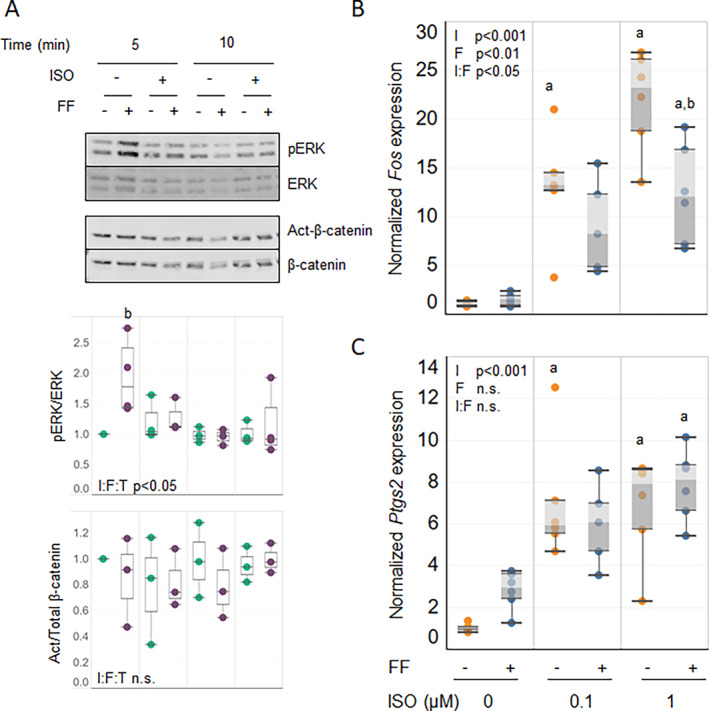
Isoproterenol (ISO) treatment selectively inhibits flow‐responsive signaling in primary outgrowth bone cells. Cells established from mice were pretreated with 0.1 or 1.0 μM ISO and subjected to fluid flow (FF). (*A*) Signaling events after flow were analyzed by Western blotting to detect ERK phosphorylation and canonical β‐catenin activation at the time points shown. Values of the normalized quantified signals are aligned below each relevant lane. Three‐way ANOVA was conducted to detect significance of ISO pretreatment (I), flow (F), and time (T) and the combination of these factors (I:F:T) on each signaling event. (*B*, *C*) After 1 hour under stationary (gold dots) or flow (blue dots) conditions, cells were analyzed for expression of mechanosensitive genes *Fos* (*B*) and *Ptgs2* (*C*) relative to β‐Actin housekeeping gene by qRT‐PCR. Two‐way ANOVA analyses were performed to detect significance of ISO pretreatment (I) and flow (F), alone and in combination (I:F), for the expression of these genes. Western blot results are representative of 3 technical replicates. qRT‐PCR data are pooled from 2 replicate runs (*n* = 6). Post hoc analyses were used (*p* < 0.05; ^a^versus no ISO; ^b^versus no flow).

To evaluate β2AR modulation of signaling and gene expression in static and mechanically stimulated bone cells, we first investigated the effects of ISO on fluid flow responses in primary outgrowth bone cells from aged mice. These cultures expressed primarily osteoblastic markers, with negligible expression of osteocytic marker genes (Supplemental Fig. S1). Fluid flow increased pERK within 5 minutes, but this effect was muted with ISO pretreatment (Fig. 3*A*). Neither flow nor ISO treatment altered levels of active β‐catenin at either time point. In these primary cultures, expression of the mechanosensitive gene *Fos* was not altered by flow alone. However, ISO treatment did cause significant elevation of *Fos* transcripts (Fig. 3*B*), which was reduced by superimposition of flow. *Ptgs2* mRNA was also significantly elevated with ISO treatment, but fluid flow did not change expression level of this gene (Fig. 3*C*).

### β2AR activation altered flow responses in osteoblastic MC3T3‐E1 cells

**Fig. 4:**
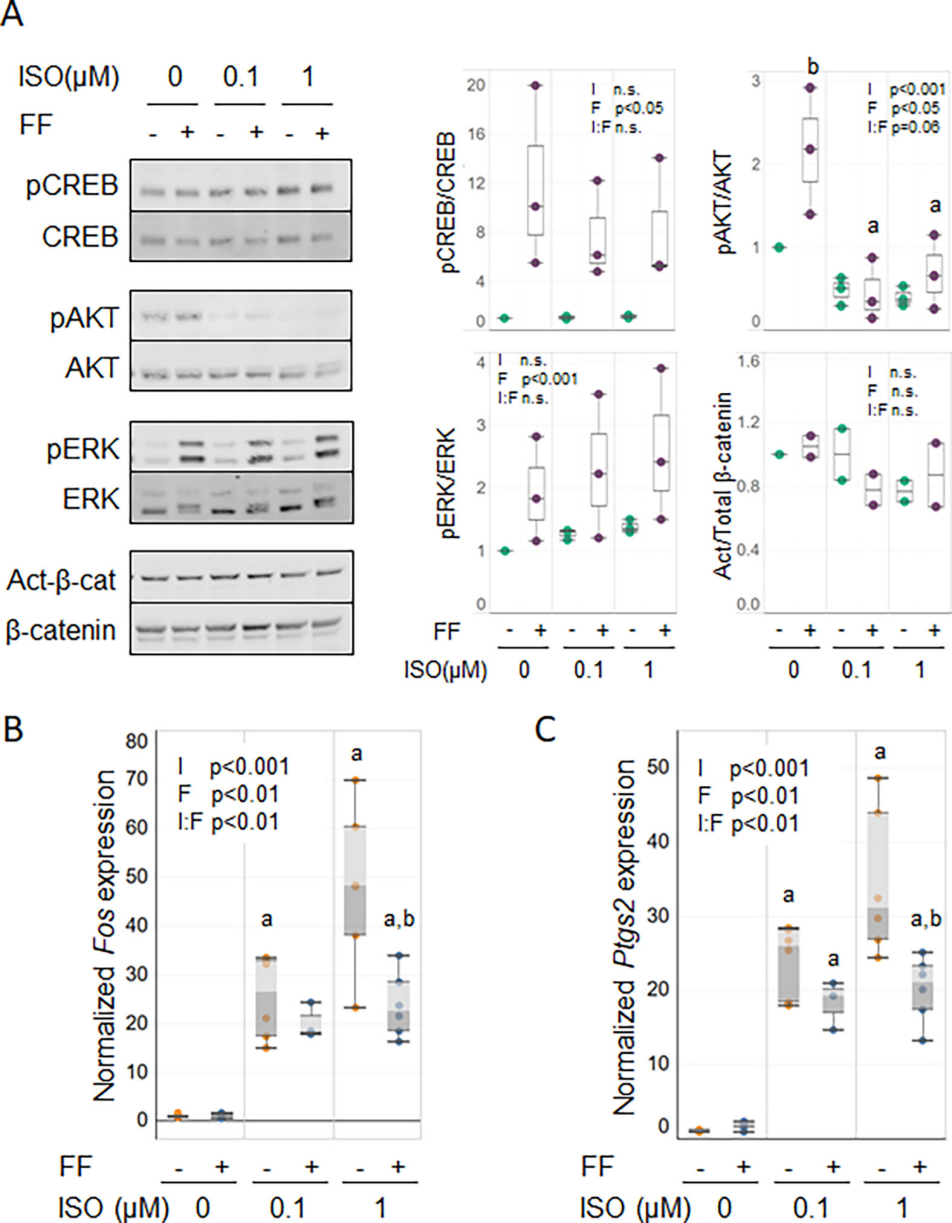
Isoproterenol (ISO) selectively inhibits flow‐induced signaling and gene expression in MC3T3‐E1 cells. MC3T3‐E1 cells were pretreated with ISO at the concentrations shown and subjected to fluid flow (FF). (*A*) Phosphorylation of CREB, AKT, ERK, and canonical β‐catenin activation after flow were analyzed by Western blotting. Quantitative analysis of the blots is shown to the right. (*B*, *C*) after 1 hour under stationary (gold dots) or flow (blue dots) conditions, expression of mechanosensitive genes *Fos* (*B*) and *Ptgs2* (*C*) was assessed by qRT‐PCR. Two‐way ANOVA analyses were performed to detect significance of ISO pretreatment (I), flow (F), and the combination of these factors (I:F), for the expression of these genes. Western blot results are representative of 3 independent experiments. qRT‐PCR data are pooled from 2 independent experiments (*n* = 6). Post hoc analyses were used (*p* < 0.05; ^a^versus no ISO; ^b^versus no flow).

To further our understanding of the effects of β2AR on mechanotransduction in the primary cultures, we turned to more homogeneous cell models. As the primaries lacked a significant osteocytic phenotype, we first evaluated the effects of fluid flow and ISO pretreatment in MLO‐Y4 osteocytic cells. We detected no significant interactions between activation of β2AR and fluid flow in either signaling or transcriptional responses (Supplemental Fig. S2), suggesting that the ISO responses observed in the primary cultures likely occurred in osteoblastic cells. Next, we treated MC3T3‐E1 cells with ISO before fluid flow exposure and investigated early flow responsive signaling pathways. Flow alone increased relative pCREB, pAKT, and pERK levels, but active β‐catenin did not change with flow (Fig. 4*A*). ISO treatment alone did not significantly alter activation of the signaling pathways tested. However, in cultures also subjected to fluid flow, the β‐agonist did inhibit the mechanical stimulation of pAKT. In contrast, levels of pCREB, pERK, and active β‐catenin in cultures exposed to ISO plus flow did not differ from those with flow alone. Consistent with the primary outgrowth cultures, neither *Fos* (Fig. 4*B*) nor *Ptgs2* (Fig. 4*C*) was elevated by fluid flow alone. However, pretreatment with ISO increased expression of both genes in the absence of flow. This elevated expression was significantly reduced when fluid flow was superimposed upon ISO pretreatment but remained elevated above the level of expression with flow alone.

### Co‐treatment of MC3T3‐E1 cells with β2AR antagonist Butax mitigated effects of ISO

**Fig. 5:**
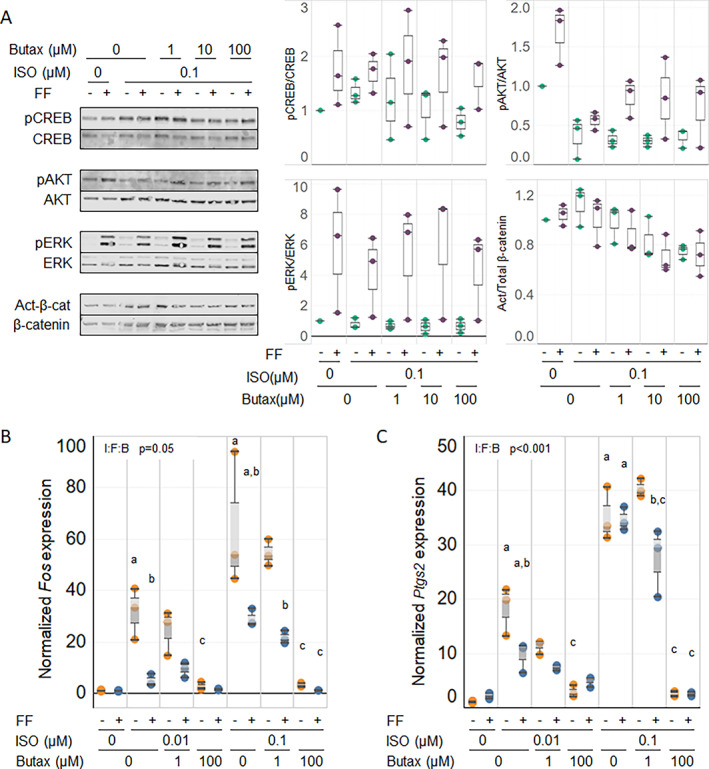
Butax co‐treatment reverses isoproterenol (ISO)‐induced effects in MC3T3‐E1 cells. MC3T3‐E1 cells were pretreated with ISO and Butax at the concentrations shown and subjected to fluid flow (FF). (*A*) Phosphorylation of CREB, AKT, ERK, and canonical β‐catenin activation after flow was analyzed by Western blotting. Quantification of the blots is shown to the right, but the unbalanced study design and small group sizes precluded statistical analysis. (*B*, *C*) After 1 hour under stationary (gold dots) or flow (blue dots) conditions, cells were analyzed by qRT‐PCR for expression of mechanosensitive genes *Fos* (*B*) and *Ptgs2* (*C*). Three‐way ANOVA analyses were performed to detect significance of ISO (I), flow (F), Butax (B), and the combination of these factors (I:F:B), for the expression of these genes. Post hoc analyses were used (*p* < 0.05; ^a^versus no ISO; ^b^versus no flow; ^c^versus no Butax). Western blot results are representative of 3 independent experiments. qRT‐PCR data are pooled from 3 independent experiments (*n* = 3–9).

To confirm that ISO effects on fluid flow‐induced signaling in MC3T3‐E1 cells were mediated via β2AR signaling, we explored co‐treatment with Butax. Qualitatively, elevation of pAKT by flow was lessened when superimposed with ISO treatment (Fig. 5*A*). As expected, concurrent treatment with Butax tended to mitigate the ISO inhibitory effect on pAKT in the presence of flow, partially restoring the flow response despite exposure of the cells to ISO. In the parallel expression analysis, co‐treatment with Butax also reversed the ISO‐induced increase of *Fos* (Fig. 5*B*) and *Ptgs2* (Fig. 5*C*) transcripts in a concentration‐dependent manner, with little effect on the flow‐induced decrease in ISO‐treated cells. Butax treatment did not alter levels of pCREB, pERK, or active β‐catenin when combined with ISO treatment, consistent with the lack of ISO effect on these signaling molecules in the earlier experiment.

## Discussion

Pretreatment of aged female mice with the β2AR‐selective antagonist Butax enhanced periosteal bone formation induced by direct skeletal loading but did not alter bone formation in contralateral tibias, suggesting that β2AR mitigation was not sufficient to enhance bone formation on its own. In vitro, the activation of β2AR by ISO treatment before fluid flow exposure differentially altered mechanically responsive signal transduction and early mechanosensitive gene expression in osteogenic cell cultures. Concurrent treatment with Butax reversed the signaling effects of ISO, supporting the role of β2AR in this response and highlighting potential mechanisms mediating Butax‐enhanced mechanotransduction in vivo.

To our knowledge, this study is the first to investigate potential βAR‐antagonist modulation as a means to enhance the minimal response of the aged skeleton to mechanical loading. Previous explorations of the sympathetic modulation of bone cell responses to skeletal loading in young adult rodents, however, provide supportive context for our observation despite superficially conflicting data. Genetic studies in single and double null mutant male mice concluded that β1AR, but not β2AR, was required to increase periosteal bone formation after mechanical stimulation.^(^ ^41^ ^)^ In that study, however, periosteal mineral apposition rate of mechanically stimulated β2AR tibias was almost twice that of wild‐type littermates, which is consistent with our findings. In β2AR KO male mice of similar age, we have observed a smaller tibial cross section and reduced cortical moment of inertia versus littermate controls, which would result in increased peak normal strain, as loading magnitude was constant across groups.^(^ ^42^ ^)^ Thus, the enhanced loading response of the β2AR‐deficient tibias observed by Pierroz and colleagues could potentially have arisen by some combination of altered tibial morphology and an enhanced responsiveness to loading. Our finding that treatment with a β2AR‐selective antagonist enhanced the bone mechanical response in C57BL/6 female mice adds support for the latter mechanism.

Our findings with Butax contrast with pharmacologic studies in young adult mice, which suggested that after reduction of sympathetic signaling by a chemical or surgical approach, treatment with the β1, β2‐antagonist propranolol (PRO) did not alter the mechanical response of cortical bone.^(^ ^43^, ^44^ ^)^ Our findings are consistent, however, with previous studies indicating that elevation of sympathetic tone may reduce the anabolic response of bone to loading. For example, sympathetic modulation of the bone mechanical response was evident after treatment of adult rats with the β2AR‐selective agonist salbutamol, which blocked an exercise‐induced increase in cortical bone formation.^(^ ^45^ ^)^ Additionally, bilateral lesion of the inner ear vestibula led to increased sympathetic outflow and a loss of bone mass in weight‐bearing bones due to reduced bone formation; this loss was prevented by PRO and by deletion of β2AR,^(^ ^46^ ^)^ consistent with the Butax effect we observed in aged mice. This parallel between elevation of sympathetic tone and anabolic effects of beta antagonists on the response of bone to mechanical stimuli may, in part, explain why the findings of the present study differ from those of previous investigations in which sympathetic tone was lowered. Other contributing factors could be aligned with our pharmacologic strategy. For example, selective affinity for β2AR and poor penetrance at the blood brain barrier^(^ ^34^ ^)^ would limit Butax effects to peripheral tissues, whereas PRO, which readily passes into the brain,^(^ ^47^ ^)^ could impact central as well as peripheral β1AR and β2AR activity.

Our detection of β2AR antagonist effects on the bone mechanical response was likely facilitated by our use of aged animals, as senescent rodents demonstrate higher sympathetic tone.^(^ ^13^, ^19^, ^46^ ^)^ Selective antagonism of β2AR also holds potential to enhance bone mechanotransduction more broadly, and this concept can be directly tested in younger adult mice. Another limitation of the present study is that Butax effects on bone mechanotransduction were not tested in male mice. Sympathetic restraint of bone mass was associated with a progressive increase in norepinephrine content of cortical bone in aging norepinephrine transporter (NET)‐deficient male mice.^(^ ^11^, ^48^ ^)^ In addition, we have observed an enhanced periosteal response to mechanical stimulation in aged male β2AR‐deficient mice.^(^ ^42^ ^)^ Thus, although sex‐related differences in sympathetic regulation have been reported,^(^ ^49^, ^50^ ^)^ the available evidence suggests that Butax treatment would also enhance the bone mechanical response in aged male C57BL/6 mice.

Butax treatment may relieve sympathetic suppression of mechanically stimulated osteoblast differentiation in vivo, as predicted by the findings of in vitro studies using PRO^(^ ^51^ ^)^ and ISO^(^ ^52^ ^)^ to manipulate β‐adrenergic activity in osteoblastic cultures. Mechanically stimulated calcium signaling is known to enhance osteoblast proliferation and differentiation through PI3K/AKT, ERK/Elk1, and CaMK/CREB signaling pathways.^(^ ^53^, ^54^, ^55^, ^56^, ^57^, ^58^ ^)^ Consistent with the Butax enhancement of the mechanical response of bone in vivo, our in vitro data demonstrate the potential for β2AR signaling to counter typical osteoblastic fluid flow responses, as ISO activation of β2AR signaling abolished flow responsive enhancement of pERK in primary cells and pAKT in MC3T3‐E1 cells, and Butax prevented the latter. Although it is well established that fluid flow shear stress can enhance expression of the early effectors *Fos* and *Ptgs2* in osteoblasts,^(^ ^59^, ^60^ ^)^ we did not see robust flow‐induced increases in expression of mechanoresponsive *Fos* and *Ptsg2* in our cells. As we have previously observed flow responsive expression of these genes under the same experimental conditions,^(^ ^38^ ^)^ this deficit may be attributable to the low serum conditions used to optimize ISO responses in this study. Although ISO treatment greatly enhanced expression of both *Fos* and *Ptgs2*, consistent with previous reports,^(^ ^61^, ^62^, ^63^ ^)^ fluid flow reduced the ISO‐induced elevation. This unexpected finding suggests that mechanotransduction may interfere with β2AR‐activated transcription in osteoblastic cells.

We found that mechanically enhanced ERK1/2 phosphorylation was inhibited by ISO treatment in primary, but not MC3T3‐E1, cultures. Our long bone osteogenic cultures from aged mice were predominately mature osteoblasts, whereas MC3T3‐E1 cells cultured in the absence of osteogenic stimuli are pre‐osteoblastic.^(^ ^64^ ^)^ This is consistent with a previous study demonstrating that juvenile mouse long bone primary cultures were at a later stage of osteoblast differentiation than neonatal calvarial cultures before in vitro differentiation.^(^ ^65^ ^)^ In another study, although mechanotransduction was comparable in primary bone cell cultures regardless of donor age or skeletal origin, neonatal cultures had a stronger biochemical response than adult cultures.^(^ ^66^ ^)^ By extension, these previous studies suggest that disparate signaling responses to the β‐agonist in our two culture models may be due to an age‐related influence on stage of osteoblast differentiation that determines biochemical responses to ISO, with the β2AR agonist blocking mechanically stimulated ERK1/2 activation in the pre‐osteoblasts but not the more mature cells. Future studies will explore this insight to determine whether β2AR expression, signaling, and interactions with mechanotransduction vary according to osteoblastic stage of differentiation.

In both osteoblasts and osteocytes, fluid flow has been reported to stabilize β‐catenin via the upstream inactivation of GSK3β, mediated by the activation of AKT.^(^ ^58^, ^67^, ^68^, ^69^ ^)^ Given the essential role of Wnt signaling in osteoblasts and our observed loss of flow‐induced pAKT with ISO treatment, it is surprising that we did not see a change in active β‐catenin levels with either flow or ISO treatment. Activation and nuclear localization of β‐catenin in response to fluid shear stress has commonly been observed in osteoblastic cells after one or more hours of stimulation^(^ ^58^, ^67^, ^70^, ^71^ ^)^ and under sufficient magnitude of shear stress.^(^ ^70^ ^)^ Thus, our short period of more modest fluid flow magnitude (2 dyn/cm^2^ versus 10 or 12 dyn/cm^2^ in the previously cited work) may not have been sufficient to replicate previous studies. It is possible that β2AR treatment could have an inhibitory effect on flow‐induced β‐catenin activation under more vigorous or prolonged flow conditions.

Induction of Fos^(^ ^72^ ^)^ and COX‐2^(^ ^73^ ^)^ expression are among the earliest activated mechanotransduction pathways in bone. Although this pattern has been reproduced in MC3T3‐E1 cells,^(^ ^59^ ^)^ we did not observe these increases in this study. In fact, exposure to shear stress dampened a positive β‐agonist effect on expression of *Fos* and *Ptgs2* expression. In this regard, our in vitro results were not directly aligned with the enhanced anabolic response to mechanical stimulation in Butax‐treated mice. A similar lack of consistency between live animal and cell culture studies has been noted in the context of connexin 43 and mechanotransduction.^(^ ^74^ ^)^ Suggested reasons for that discrepancy included the use of two‐dimensional cell cultures and the experimental focus on only one mechanical signal,^(^ ^74^ ^)^ which would both apply here. The inconsistency may also relate to the mixture of mechanically responsive cell populations in skeletal tissue (osteobasts, osteocytes, and stromal cells) versus the predominantly osteoblastic cells in vitro. Finally, in addition to β2AR, mouse osteoblast lineage and stromal cells express α_1A_AR and α_1D_AR, and MC3T3‐E1 cells express α_2A_AR as well (reviewed by Elefteriou^(^ ^75^ ^)^). Thus, whereas β2AR is presumably the predominant responder to ISO in the cell culture studies, the α‐ARs in bone cells in vivo can also be activated by the endogenous catecholamine norepinephrine.

In summary, the anabolic response to mechanical loading of mouse tibias was enhanced at the periosteum by the β2‐antagonist Butax, but bone formation on the contralateral limb was unaffected. Butax treatment was associated with increased mineralizing surface but not mineral apposition rate. This result suggests that sympathetic output primarily reduces activation of the osteoblastic cell population by mechanical stimulation, with less impact on osteoblast function. The in vitro responses to flow and ISO were complex, varying with the signaling pathway investigated and the cell model employed. In sum, however, our data provide clear evidence for negative interactions between mechanically stimulated and active β2AR signaling. These findings support further explorations into the potential clinical benefit of selective β2AR antagonism as a therapeutic adjuvant to exercise.

## Conflicts of Interest

The authors have no potential or real conflicts of interest to disclose.

## Author Contributions

**Leah Worton:** Data curation; formal analysis; investigation; writing – original draft; writing – review and editing. **Sundar Srinivasan:** Conceptualization; data curation; formal analysis; funding acquisition; investigation; supervision; validation; writing – original draft; writing – review and editing. **DeWayne Threet:** Investigation; validation. **Brandon J Ausk:** Validation; visualization; writing – review and editing. **Phillipe Huber:** Visualization; writing – review and editing. **Ronald Kwon:** Conceptualization; funding acquisition; writing – review and editing. **Steven D. Bain:** Conceptualization; funding acquisition; writing – review and editing. **Ted S. Gross:** Conceptualization; supervision; validation; writing – original draft; writing – review and editing. **Edith Gardiner:** Conceptualization; funding acquisition; project administration; supervision; validation; writing – original draft; writing – review and editing.

## Supporting information


**Appendix S1.** Supporting information

## Data Availability

Data will be made available on request from the authors.
