# Supplementary material for: Beta 2 Adrenergic Receptor Selective Antagonist Enhances Mechanically Stimulated Bone Anabolism in Aged Mice
Source: JBMR Plus. 2022 Dec 27;7(2):e10712. doi: 10.1002/jbm4.10712 (PMC9893264; doi:10.1002/jbm4.10712)
Supplement: Supplementary file 1 — Appendix S1. Supporting information [file JBM4-7-e10712-s001.pdf]

Table S1. PCR primer sequences

Target	Forward (5'→3')	Reverse (5'→3')
β-actin	TCACCCACACTGTGCCCATCTACGA	CAGCGGAACCGCTCATTGCCAATGG
Ppargc1a	CAGAGTCACCAAATGACCCCAA	CCCTGAGGACTTGCTGAGTT
Ucp1	TCTGCATGGGATCAAACCCC	ACAGTAAATGGCAGGGGACG
Fos	CTCCCGTGGTCACCTGTACT	TTGCCTTCTCTGACTGCTCA
Ptgs2	GCTGTACAAGCAGTGGCAAA	CCCCAAAGATAGCATCTGGA
Runx2	AAGTGCGGTGCAAACTTTCT	TCTCGGTGGCTGGTAGTGA
Dlx5	CCCAAGCATCCGATCCGGCG	GCCGTTCACGCCGTGGTACT
Sp7	CCCTTCTCAAGCACCAATGG	AGGGTGGGTAGTCATTTGCATAG
Alpl	GCCCTCTCCAAGACATATA	CCATGATCACGTCGATATCC
Ibsp	CGGTTTCCAGTCCAGGGAGGC	TTGGGCAGTTGGAGTGCCGC
Col1a1	AATGGCACGGCTGTGTGCGA	AACGGGTCCCCTTGGGCCTT
Spp1	TGCACCCAGATCCTATAGCC	CTCCATCGTCATCATCATCG
Mgp	ATGAAGAGCCTGCTCCCTCT	ATATTTGGCTCCTCGGCGCT
Bglap2	CTCTGTCTCTCTGACCTCACAG	GGAGCTGCTGTGACATCCATAC
Pdpn	GCCAGTGTTGTTCTGGGTTT	AGAGGTGCCTTGCCAGTAGA
Dmp1	CACCACCACCACCCACGAACA	GGCCTCTGTCGTAGCCCAGC
Phex	GATTGAGGGTGTTCGCTGGGCC	TTGCGGGTTTGCAGTACGTTGCC
Fgf23	AGTCGGTTCAGCCCACGTAGAGGA	TGAATAGCGGTGCCTGGCTGGA
Mepe	ATGCCCAGAGACTAAGCCCGAA	TTCATTCGGCATTGGTGCCGC
Sost	GCCGGACCTATACAGGACAA	CACGTAGCCCAACATCACAC

Table S2. Antibodies

Epitope	Source	Host Species	Dilution	Details
p-CREB	Millipore; 06-519	Rabbit	1:1000	5% skim milk/TBS tween, 4C overnight
CREB	Cell Signaling Technology; 9104	Mouse	1:1000	5% skim milk/TBS tween, 4C overnight
pERK	Cell Signaling Technology; 9101	Rabbit	1:1000	5% skim milk/TBS tween, 4C overnight
ERK	Cell Signaling Technology; 4696	Mouse	1:1000	5% skim milk/TBS tween, 4C overnight
ERK	Cell Signaling Technology; 4695	Rabbit	1:1000	5% skim milk/TBS tween, 4C overnight
pAKT	Cell Signaling Technology; 4051	Mouse	1:1000	5% skim milk/TBS tween, 4C overnight
AKT	Cell Signaling Technology; 9272	Rabbit	1:1000	5% skim milk/TBS tween, 4C overnight
Active β-catenin	Cell Signaling Technology; 8814	Rabbit	1:1000	5% BSA/TBS tween, 4C overnight
β-catenin	BD Biosciences; 610153	Mouse	1:2000	5% BSA/TBS tween, 4C overnight

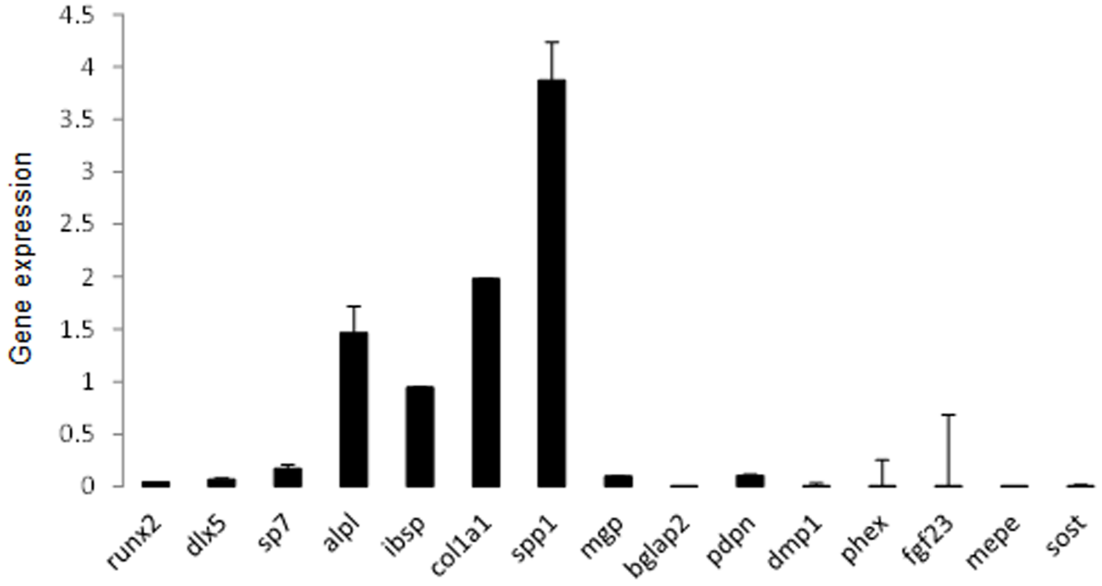

Figure S1. Primary bone cell cultures from senescent (22 Mo) C57BL/6J female mice were analyzed by qRT-PCR for expression of osteoblastic and osteocytic markers (ordered from left to right according to approximate temporal expression during differentiation). Data are pooled from 5 independent experiments and graphed as mean + SE (n=9-15).

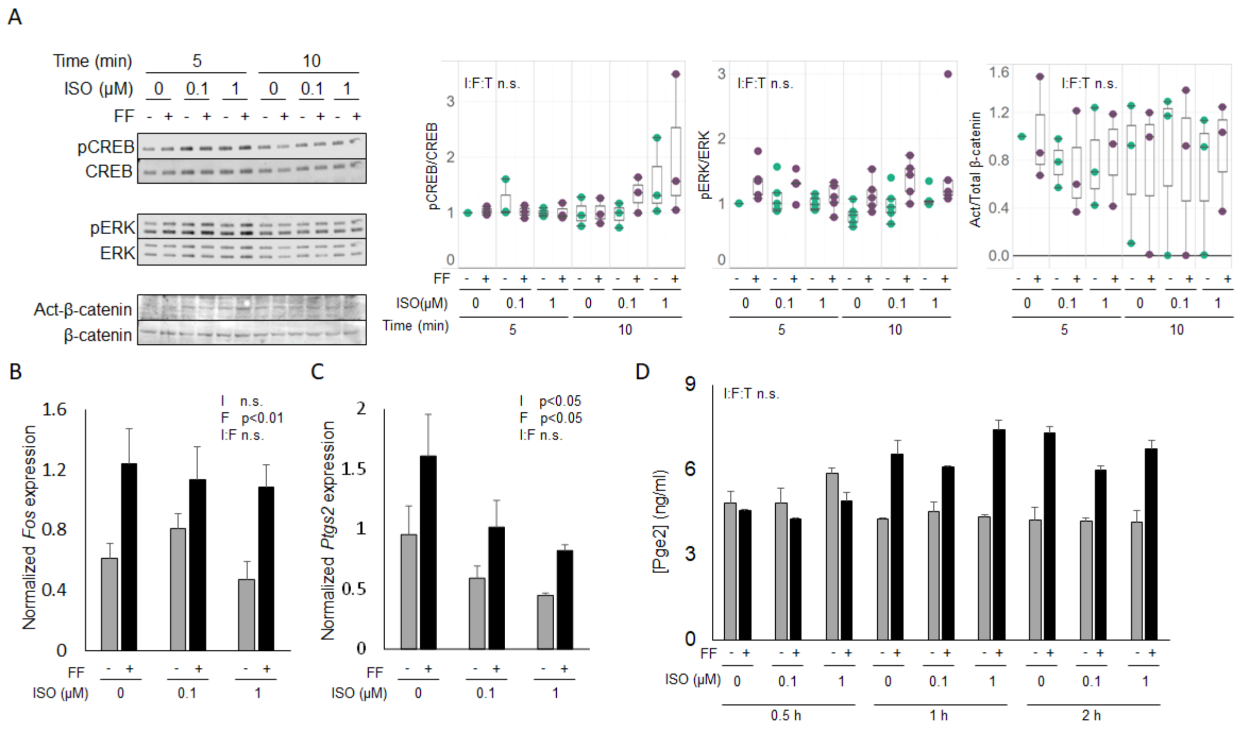

Figure S2. MLO-Y4 cultures treated with ISO prior to fluid flow exposure did not exhibit altered flow induced phosphorylation patterns of CREB or ERK, and activate β-catenin levels were low in all conditions (A). Mechanoresponsive genes *Fos* (B) and *Ptgs2* (C) were stimulated by flow but there was no ISO effect on flow responsive expression of either gene. Similarly, although prostaglandin PGE2 release into culture medium at 1 h and 2 h was stimulated by flow, there was no interaction between ISO and flow over different time points for PGE2 release (D). The reason for this lack of an ISO effect is not obvious. Osteocytic expression of β2AR has been detected *in vivo*[S1] and in MLO-Y4 cells (data not shown). Furthermore, norepinephrine transporter in osteocytes appears to reduce the anti-anabolic influence of sympathetic nerves *in vivo.*[11,50] It may be informative to investigate mechanotransduction under altered β2AR activity in other osteocytic cell models.

Method: MLO-Y4 osteocyte-like cells, derived from murine long bones, were provided by Dr. Lynda Bonewald and cultured as previously described to maintain the dendritic phenotype.[S2] Cells were seeded at 2.5x10^4 cells per well in 2ml media (α-MEM with 2.5% FBS and 2.5% calf serum on collagen coated plates). At 72h cells were treated

with ISO and exposed to fluid flow as described for the MC3T3-E1 studies. Prostaglandin PGE2 ELISA (ENZO, ADI-900-001) was performed using media collected after flow (30min, 1h, 2h).

Supplemental references

S1. Asada N, Katayama Y, Sato M, Minagawa K, Wakahashi K, Kawano H, et al. Matrix-embedded osteocytes regulate mobilization of hematopoietic stem/progenitor cells. Cell Stem Cell. Jun 6 2013;12(6):737-47.

S2. Kato Y, Windle JJ, Koop BA, Mundy GR, Bonewald LF. Establishment of an osteocyte-like cell line, MLO-Y4. J Bone Miner Res. Dec 1997;12(12):2014-23.
